# A Mindfulness-Based Intervention for Binge Eating, Self-Compassion, and Mindfulness in Brazilian Women With Weight Regain After Metabolic and Bariatric Surgery: a Pilot Feasibility Study

**DOI:** 10.1007/s11695-026-08520-9

**Published:** 2026-02-23

**Authors:** Ana Flávia de Sousa Silva, João Henrique Fabiano Motarelli, Geórgia das Graças Pena, Fernanda Rodrigues de Oliveira Penaforte, Camila Cremonezi Japur

**Affiliations:** 1 Universidade de São Paulo, São Paulo, Brazil; 2 Universidade Federal do Triângulo Mineiro, Uberaba, Brazil; 3 Instituto Consciência Alimentar, São Paulo, Brazil; 4 Universidade Federal de Uberlândia, Uberlândia, Brazil; 5 Núcleo de Estudos, Pesquisa e Extensão em Obesidade e Comportamento Alimentar - NEPOCA, Ribeirão Preto, Brasil

**Keywords:** Weight regain, Bariatric surgery, Obesity, Mindfulness

## Abstract

**Introduction:**

From a biomedical perspective, metabolic and bariatric surgery can effectively treat obesity. However, traditional strategies (diet and exercise) to prevent weight regain have not been fully effective, as they may help some individuals maintain postoperative weight loss, but approximately 30% of patients still regain weight after surgery. This highlights the need to explore alternative approaches, including mindfulness-based interventions. This study aims to evaluate the feasibility and describe the effects of a mindfulness-based intervention on behavioral and anthropometric outcomes in women with weight regain after metabolic and bariatric surgery. Methods: A pre-post feasibility pilot study was conducted with 10 women who had regained ≥ 15% of the achieved weight loss. The intervention combined Mindfulness-Based Eating Awareness Training with elements of the Mindful Self-Compassion program. Feasibility outcomes included adherence, attendance (overall and among completers), recruitment rate, data completeness, and informal facilitator feedback. Behavioral (binge eating, self-compassion, mindfulness) and anthropometric measures (weight and BMI) were assessed at three time points: baseline (T1), post-intervention (T2), and 3-month follow-up (T3).

**Results:**

Feasibility indicators revealed low adherence (60% dropout), low overall attendance (41.7%), high attendance among completers (87.5%), moderate recruitment rate (38.5%), and low data completeness (40.0%). Challenges in engagement were associated with expectations of diet-based approaches. Among completers, improvements were observed in binge eating (22.7 ± 5.4 to 11.3 ± 2.5), “Isolation” (2.6 ± 0.4 vs. 2.3 ± 0.4), “Over-Identification” (3.3 ± 0.5 vs. 2.8 ± 0.2), and “Mindfulness” (3.1 ± 0.3 vs. 3.5 ± 0.4) subscales of the Self-Compassion Scale. From baseline to the 3-month follow-up, we observed improvement in “Mindfulness” (3.1 ± 0.3 vs. 3.9 ± 0.2), “Over-Identification” (3.3 ± 0.5 vs. 2.8 ± 0.3), and “Nonreactivity to Inner Experience” (19.5 ± 1.6 vs. 22.2 ± 2.2). Body weight remained stable throughout the intervention period (82.1 ± 4.0 vs 81.7 ± 4.0) and reduced at 3-month follow-up (79.8 ± 3.7).

**Conclusion:**

Although not feasible in its current format, the intervention showed preliminary benefits, indicating the need for protocol refinement and a new feasibility evaluation before moving to larger trials.

## Introduction

Metabolic and bariatric surgery (MBS) is well established as an effective treatment for achieving substantial and sustained weight loss, as well as for improving obesity-related comorbidities, with patients losing around 40% of their initial body weight within the first postoperative year [1]. However, despite its effectiveness, approximately 30% of patients experience insufficient weight loss or subsequent weight regain within one to two years after surgery [2], underscoring the need for complementary behavioral strategies to enhance long-term outcomes.

Disordered eating behaviors, physiological compensatory mechanisms, and eating-related psychopathology, such as dietary disinhibition, heightened hunger (defined as both a physiological drive to eat, resulting from compensatory hormonal and neuroendocrine changes that counteract the initial appetite-suppressing effects of surgery, and the subjective perception of intensified hunger reported by patients), impulsivity, and binge eating, are among the main factors contributing to weight regain [3, 4].

Binge eating symptoms are not necessarily a postoperative complication but rather a pre-existing or persistent eating behavior that may remain unrecognized or untreated after surgery. Importantly, although binge eating symptoms may decrease in the early postoperative period due to surgical restriction and intensive clinical follow-up, evidence suggests that they may reemerge or change in form over time, often shifting from large eating episodes to more subtle patterns of loss of control [5, 6]. In this context, surgery alone does not modify or eliminate binge eating behaviors, which often persist in the absence of adequate psychological treatment [3, 4]. This highlights the importance of integrating behavioral and psychological interventions into postoperative care to support long-term weight management. Nevertheless, these symptoms can significantly influence postoperative outcomes, as loss of control over eating is directly associated with weight regain [3, 4].

Although binge eating symptoms represent the core feature of Binge Eating Disorder (BED), as defined in the DSM-5, it may also manifest as an isolated behavior or within the context of other psychiatric conditions, occurring at subthreshold or low-frequency levels, categorized as Other Specified Feeding or Eating Disorder (OSFED- BED of low frequency and/or limited duration), or as manifestations of major depression or bipolar disorder [4, 7]. In the present study, the term “binge eating” is used to denote self-reported episodes of loss of control over eating, without necessarily implying a formal diagnosis of a psychiatric condition.

Moreover, it is important to distinguish binge eating, characterized by perceived loss of control and consumption of an objectively large amount of food, from emotional eating, which refers to eating in response to affective states, often without loss of control [8]. Both are maladaptive eating patterns linked to emotional dysregulation and impulsivity, constructs that mindfulness- and self-compassion-based interventions specifically aim to address [9–11].

Weight regain after MBS may compromise metabolic outcomes, as improvements in metabolic indicators are closely tied to weight loss. Thus, postoperative weight regain warrants close clinical attention [12, 13]. Importantly, qualitative research demonstrates that weight regain carries not only physiological consequences, but also significant psychosocial repercussions. In Silva et al. [14], women reported that the return of weight disrupted the sense of renewal and transformation initially associated with surgery, often interpreting this experience as a setback in their personal journey. Barbosa et al. [15] identified negative emotional repercussions such as shame, frustration, and self-blame, with some individuals avoiding follow-up care or social exposure due to perceived judgment. These findings highlight that postoperative trajectories are shaped by both physiological mechanisms and the negative emotional repercussions of weight regain, including subjective experiences and internalized stigma.

Conventional treatments such as diet and physical activity have shown limited effectiveness in addressing long-term weight regain, as they primarily focus on biological and prescriptive aspects, with little emphasis on coping strategies for stress management or on the behavioral and emotional dimensions of eating [16, 17]. In this context, mindful eating–based interventions have emerged as promising, showing positive effects on eating behavior, self-esteem, reductions in binge eating episodes, and improvements in mindful eating skills [18, 19]. Regular mindfulness practice has been shown to reduce the activity of brain regions associated with rumination and self-criticism, such as the medial prefrontal cortex, posterior cingulate cortex, precuneus, hippocampus, and inferior parietal lobe. This neural modulation contributes to the reduction of negative thoughts and feelings of guilt [20], which are frequently linked to binge eating episodes, characterized by the consumption of a large amount of food within a discrete period (e.g., 2 h), accompanied by a sense of loss of control over eating [7]. Moreover, mindfulness can foster self-compassion and enhance perceived control over eating behavior [21].

The Mindfulness-Based Eating Awareness Training (MB-EAT) is an internationally recognized mindful eating protocol developed through multiple clinical trials to address binge eating disorder in individuals with obesity. It incorporates mindfulness-based practices aimed at helping individuals gain control over their responses to emotional states, make conscious food choices by enhancing awareness of hunger and satiety cues, and foster self-acceptance. Overall, MB-EAT seeks to increase awareness of internal and external eating triggers, interrupt dysfunctional cycles of binge eating, self-recrimination, and dietary restriction, and restore the natural physiological processes of eating regulation—ultimately promoting healthier eating patterns in quantity and quality [22, 23].

Current evidence supports the benefits of MB-EAT in improving quality of life by enhancing individuals' perceived control over eating behavior [24]. Given that many patients who undergo MBS often experience challenges with emotional regulation and eating dysregulation, MB-EAT may represent a promising intervention for this population. Chacko et al. [25] applied the MB-EAT protocol in adults 1 to 5 years post- MBS and found the intervention highly acceptable and effective in reducing emotional eating. Similarly, Wnuk et al. [26] conducted a study with post- MBS women within the same postoperative window. They reported significant reductions in depression, improvements in emotional regulation, and reductions in emotional and binge eating. These findings underscore the feasibility of MB-EAT for addressing both disordered eating behaviors and associated mental health symptoms in individuals who have undergone MBS.

Self-compassion has emerged as a protective psychological resource for individuals experiencing weight regain after MBS. A previous study showed that higher levels of preoperative self-compassion predicted lower levels of depression, greater quality of life, higher body image satisfaction, and better self-efficacy for eating behavior control after MBS [27]. By promoting a more accepting and supportive response to perceived setbacks, self-compassion may help mitigate the negative psychological impact of weight regain and facilitate reengagement with health-promoting behaviors [28]. The Mindful Self-Compassion (MSC) program is an 8-week structured intervention to cultivate self-compassion through experiential learning (versus self-judgment) explicitly. The program is grounded in mindfulness and compassion-based approaches and teaches both formal (meditation-based) and informal (everyday life) self-compassion practices, focusing on developing self-kindness, a sense of common humanity, and mindful awareness of suffering (versus over-identification) [29].

Based on the rationale presented, integrating the MB-EAT intervention with components of the MSC program may offer meaningful benefits for individuals who have undergone bariatric surgery and experienced weight regain. However, as far as we know, only two studies using MB-EAT have included individuals who have undergone MBS [25, 26], and none have implemented the complete MSC program in this population. The scarcity of studies on this population motivated the present investigation, particularly given the absence of published research involving a Brazilian sample. This is especially relevant considering Brazil performs the second-highest number of MBS worldwide, with women accounting for approximately 70% of these procedures [30]. Furthermore, weight regain and associated maladaptive eating behaviors, such as binge eating and emotional eating, tend to affect women more frequently and intensely, both physiologically and psychosocially, making this subgroup particularly vulnerable to adverse postoperative outcomes [31]. Notably, most studies on binge eating have been conducted with female samples, reflecting both the higher prevalence of these behaviors among women and the gender-specific factors that influence disordered eating patterns.

The present study's findings may provide novel evidence to support the clinical management of this high-risk population by promoting more effective and personalized therapeutic strategies. Such strategies have the potential to reduce healthcare costs, both in outpatient and inpatient settings, and to prevent complications associated with weight regain. Accordingly, this study aimed to evaluate the feasibility and describe the effects of a mindfulness-based intervention primarily structured around Mindfulness-Based Eating Awareness Training (MB-EAT), incorporating selected components of the Mindful Self-Compassion (MSC) program. Given the pilot design of this study, it focused on providing process outcomes rather than concluding on statistical significance.

## Methods

### Study Design

This is a pre-post feasibility pilot study with a protocol of mindfulness-based intervention, following the recommendations of the CONSORT pilot and feasibility trials checklist [32]. This study was approved by the Ethics Committee for Research Involving Human (36063120.4.0000.5440) and by the National Commission for Ethics in Research – CONEP (4.446.456).

The outcomes (levels of binge eating, self-compassion, mindfulness, weight, and body mass index) were evaluated at three time points: baseline (T1), post-intervention (T2), and 3-month follow-up (T3).

### Participants

Eligible participants were adult women who had undergone bariatric surgery within the past five years (with a minimum of two years post-surgery), self-reported episodes of binge eating, and experienced post- MBS weight regain.

Weight regain was defined as the progressive increase in body weight after an initially satisfactory reduction, corresponding to the recovery of ≥ 15% of the previously achieved weight loss [33].

Only women were included, given that approximately 70% of bariatric procedures in Brazil are performed in this population [30]. Furthermore, studies that reported a high frequency of binge eating in individuals who underwent MBS were predominantly conducted with women, reflecting both the higher prevalence of these behaviors among women and the gender-specific factors that influence disordered eating patterns [28].

The definition of the Diagnostic and Statistical Manual of Mental Disorders- Fifth Edition (DSM-5) [7] was presented to the participants to guide the self-report of binge eating episodes. Although patients reported difficulty consuming large amounts of food, since anatomical changes induced by the surgical procedure result in limited gastric capacity, they usually experience a feeling of loss of food control associated with subjective feelings of guilt [34, 35].

Exclusion criteria included participants who were currently dieting or taking any medication to lose weight intentionally. At the time of data collection (June 2022 – February 2023), GLP-1 analogues were not yet in routine clinical use in Brazil to treat obesity; therefore, no participants were excluded. Pregnant or breastfeeding women were also excluded, as well as those diagnosed with a severe psychiatric condition (e.g., schizophrenia, acute‐phase depression, alcohol or drug abuse) or in active suicidal ideation, those who used psychiatric medications, and those who practiced meditation regularly. All exclusion criteria were assessed by self-reporting.

Participants were recruited through online advertising on *Instagram*® (*n* = 26). Eligible participants (*n* = 16) attended an introductory session to discuss the group's objectives, motivation, and the importance of commitment to the intervention sessions. All participants received a Redcap® link containing the informed consent form and self-completed questionnaires to assess the outcomes. At this time, 10 participants completed and began the intervention. Although there is no strict consensus on sample size for pilot randomized controlled trials (RCTs), general recommendations suggest including 10 to 40 participants to assess feasibility. This recommendation, as proposed by Hertzog et al. [36], was used as the basis for our sample size estimation.

To characterize the sample, the following data were collected: age, profession, education, marital status, monthly income (expressed by one minimum wage = R$1302.00), time since surgery, surgical technique, minimum weight after bariatric surgery, and post-surgery weight regain.

### Mindfulness-Based Intervention

**Chart 1:**
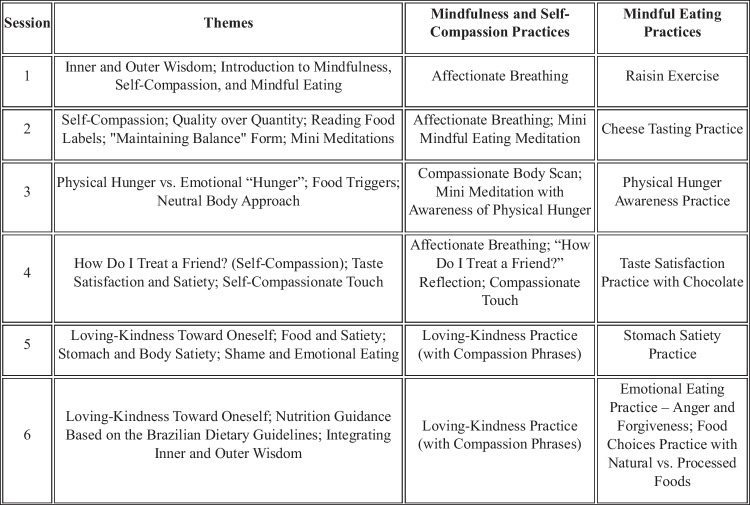
Structure of the adapted MB-EAT/MSC intervention protocol

The intervention was primarily based on Mindfulness-Based Eating Awareness Training (MB-EAT) [22], with key components from the Mindful Self-Compassion (MSC) program integrated to complement its core structure [29], along with the substitution of some foods used in the mindful eating sessions. This adapted intervention is referred to as MB-EAT/MSC for this study. It was delivered in a group format and comprised 12 sessions: 9 weekly, one biweekly, and two monthly. The structure of the sessions is presented in Chart 1.

The intervention was delivered remotely via Zoom® between June 2022 and February 2023. A qualified, experienced instructor (JHFM) led all sessions with professional training. Throughout the intervention, participants received audio guides containing formal practices of mindfulness, self-compassion, and mindful eating, along with a manual of instructions for use via the WhatsApp application.

### Feasibility Outcomes

Feasibility was assessed through adherence, attendance (overall and among completers), recruitment rate, data completeness, and informal facilitator feedback. These outcomes were evaluated according to criteria established in previous studies [37–40].

Adherence to mindfulness sessions: This domain assesses the proportion of participants who completed the intervention. It was considered adequate when the participant attended at least 9 of the 12 sessions, equivalent to 75% of the minimum attendance required [39]. Attendance: This domain assesses the frequency with which participants engaged in the scheduled intervention sessions. Average attendance was calculated for the overall sample and participants who completed the intervention, defined as those who attended at least 75% of the sessions (i.e., 9 out of 12). Recruitment: This domain assesses the proportion of individuals who, after being approached, met eligibility criteria and agreed to participate in the study. The recruitment rate was adequate when ≥ 80% of eligible individuals agreed to participate in the study [37, 39]. Data completeness: This domain assesses the proportion of participants who provided complete data at pre- and post-intervention time points. Data completeness was acceptable when ≥ 85% of participants provided complete data at pre- and post-intervention assessments [38, 40]. Informal facilitator feedback: Informal feedback from facilitators was based on their perceptions and experiences throughout the delivery of the intervention. This included reflections on participant engagement, perceived challenges in implementation, and considerations for potential improvements in future protocol applications [37, 40].

### Measures

Binge eating was measured using the Binge Eating Scale (BES), a self-administered instrument translated and adapted into Brazilian Portuguese [41]. This unidimensional scale contains 16 items, each presenting between 3 and 4 response alternatives. The responses are organized in ascending order in terms of severity. Each alternative receives a score ranging from 0 to 3 points, according to the severity level of the behavior described. An exception applies to items 4 and 16, for which response options are scored from 0 to 2 points. The total score is obtained by summing the points attributed to each item, ranging from 0 to 46, with higher scores indicating greater binge eating severity.

Self-compassion was measured by the Self-Compassion Scale, a self-report questionnaire translated and adapted to Brazilian Portuguese [42]. The scale consists of 26 items organized into six subscales that assess the following dimensions: (1) Self-Kindness, (2) Self-Judgment, (3) Sense of Common Humanity, (4) Isolation, (5) Mindfulness, and (6) Over-Identification. All items are answered on a 5-point Likert scale (1 = almost never; 5 = almost always). Items that reflect negative aspects of self-compassion (i.e., Self-Judgment, Isolation, and Over-Identification) are reverse-scored. The overall self-compassion score is computed as the mean of all item scores, with higher values indicating greater self-compassion.

Mindfulness levels were assessed using the Five Facets of Mindfulness Questionnaire (FFMQ-BR), which has been translated and adapted to Brazilian Portuguese [43]. It is composed of 39 self-report items organized into seven facets. All items are answered on a Likert-type scale from 1 (never or very rarely true) to 5 (very often or always true). The maximum score a participant can obtain on the FFMQ-BR total score is 195 points, and the minimum is 39 points, indicating the highest and lowest levels of mindfulness, respectively. For the facets, the maximum and minimum scores are as follows: (1) Non-judging of inner experience (Max. 40, Min. 8), (2) Acting with awareness – automatic pilot (Max. 25, Min. 5), (3) Observing (Max. 35, Min. 7), (4) Describing – positive formulation (Max. 25, Min. 5), (5) Describing – negative formulation (Max. 15, Min. 3), (6) Non-reactivity to inner experience (Max. 40, Min. 8), and (7) Acting with awareness – distraction (Max. 15, Min. 3).

Participants self-reported weight (in kilograms) and height (in meters). Body Mass Index (BMI) was calculated as weight divided by height squared (kg/m^2^) [44].

### Statistical Analysis

Adherence, attendance, recruitment rate, and data completeness were assessed to address the feasibility of implementing a mindfulness-based intervention. The distribution of variables was assessed using the Shapiro–Wilk test to guide appropriate summary measures. Categorical variables are presented as absolute and relative frequencies. Continuous variables are described using means and standard deviations for normally distributed data, or medians and interquartile ranges for skewed distributions. Given the feasibility nature of this study, no inferential statistical tests were conducted. Instead, descriptive statistics results from Generalized Estimating Equations (Mean ± Standard Error) explored potential trends and changes over time across the repeated measurement points. All analyses were performed using IBM® SPSS Statistics, version 23.

## Results

**Fig. 1:**
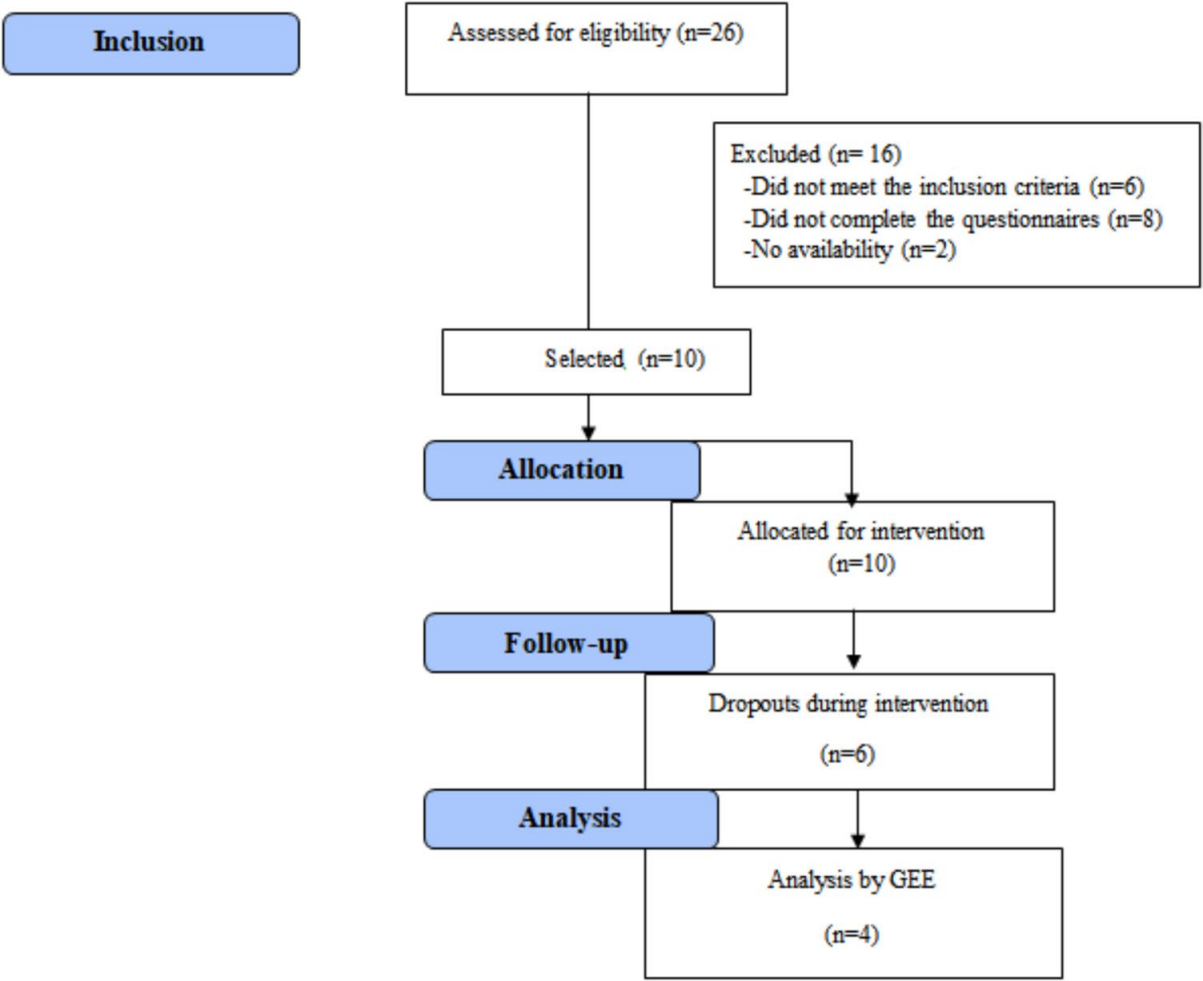
Participant flow diagram

Twenty-six participants were recruited, and after eligibility analysis, ten participants began the intervention (Fig. 1). The sample comprised cisgender women with a mean age of 41 years (± 6.3). The majority (60%) were married, had a high level of education (60%), and had a family income above three minimum wages. The only comorbidity reported by 3 of the 10 participants was Type 2 Diabetes Mellitus before surgery, but after the surgical procedure, these participants achieved remission of the disease. None declared a previous diagnosis of eating disorders. The subgroup of participants who completed the study consisted exclusively of cisgender women, with a mean age of 45 years (± 5.0). Most were married (75%), had completed an undergraduate degree (75%), and reported a household income greater than three times the minimum wage (Table 1).

**Table 1:** Socio-demographic and clinical data of the sample. Ribeirão Preto- SP, Brazil—2023

Variables	Participants (*n* = 10)	Completers (*n* = 4)
Mean (± SD) or n (%)		Mean (± SD) or *n* (%)
Age (years)	41 (± 6.3)	45 (± 5.0)
Education		
Undergraduate level	4 (40)	3 (75)
Postgraduate level	6 (60)	1 (25)
Monthly Income (Minimum wage)		
Up to 2	2 (20)	1 (25)
From 3 to 5	3 (30)	2 (50)
From 6 to 10	4 (40)	0
Above 10	1 (10)	1(25)
Marital Status		
Single	3 (30)	1 (25)
Married	6 (60)	3 (75)
Divorced	1 (10)	0
Surgical technique		
Bypass	7(70)	3 (75)
Sleeve	3(30)	1 (25)
Surgery time (years)	4 (± 1.3)	4.5 (± 1.5)
Minimum post-surgery weight (kg)	70.3 (± 12.9)	65.0 (± 4.0)
Post-surgery weight regain (%)	32.3 (± 15.5)	36.4 (± 9.4)

Adherence to the intervention was limited: of the 10 participants enrolled, only 4 completed all stages of the study, resulting in a dropout rate of 60%. Among the six participants who withdrew, three cited work-related demands, one withdrew due to the need to care for a sick family member, and two did not provide a justification. Notably, most dropouts occurred early in the intervention, with four participants discontinuing in the first week and two in the second week. Attendance among those who completed the program (at least 9 out of 12 sessions) was high, with an average session attendance of 87.5%. Specifically, one participant attended all 12 sessions (100%), one attended 11 (91.7%), one attended 10 (83.3%), and one attended nine sessions (75.0%). However, considering the full initial sample, the average overall attendance was 41.7%. The recruitment rate was 38.5%, as 10 of the 26 initially approached met the eligibility criteria and agreed to participate. Data completeness was 40.0%, as only four participants provided complete assessment data at pre- and post-intervention time points.

In this sense, the feasibility findings point to areas of eating disorders or emotional dysregulation, especially after surgery, struggle more to engage in programs requiring self-reflection or emotional exposure, focused on diet or medication, rather than a protocol grounded in mindfulness and self-compassion practices. Among those who completed the study, however, positive experiences were reported, particularly regarding improvements in eating behaviors and the development of self-compassion.

Preliminary results from baseline to post-intervention among completer participants indicated potential improvements in binge eating (22.7 ± 5.4 vs. 11.3 ± 2.5), as well as in the “Isolation” (2.6 ± 0.4 vs. 2.3 ± 0.4), “Over-Identification” (3.3 ± 0.5 vs. 2.8 ± 0.2), and “Mindfulness” (3.1 ± 0.3 vs. 3.5 ± 0.4) subscales of the Self-Compassion Scale. Improvements were also observed in the “Acting with Awareness – Distraction” subscale of the FFMQ-BR (9.0 ± 1.7 vs. 7.3 ± 0.6). From baseline to the 3-month follow-up, further potential benefits were observed in the “Mindfulness” (3.1 ± 0.3 vs. 3.9 ± 0.2) and “Over-Identification” (3.3 ± 0.5 vs. 2.8 ± 0.3) subscales of the Self-Compassion Scale, as well as in the “Nonreactivity to Inner Experience” subscale of the FFMQ-BR (19.5 ± 1.6 vs. 22.2 ± 2.2). These results are presented in Table 2. No changes in weight (82.1 ± 4.0 vs. 81.7 ± 4.0) or BMI (31.1 ± 1.3 vs. 31.1 ± 1.4) were observed from baseline to post-intervention among completers; however, reductions were noted at the 3-month follow-up in both weight (79.8 ± 3.7) and BMI (30.5 ± 1.3).

**Table 2:** Behavioral outcomes throughout the intervention in all participants and completers. Ribeirão Preto- SP, Brazil – 2023

	All participants (*n* = 10)	Completers (*n* = 4)		
Variables/Instruments	T1	T1	T2	T3
	Baseline Mean (± EP)	Baseline Mean (± EP)	Pos-Intervention Mean (± EP)	3-month follow-up Mean (± EP)
Binge eating/BES	25.4 (**± **2.5)	22.7 (± 5.4)	11.3 (**± **2.5)	16,2 (**± **2.2)
Self-compassion/SCS	2.6 (**± **0.2)	3.1 (± 0.5)	3.2 (**± **0.2)	3.1 (**± **0.2)
Self-kindness	2.8 (**± **0.3)	3.4 (± 0.5)	3.3 (**± **0.2)	3.0 (**± **0.3)
Self-judgement	3.6 (**± **0.3)	3.0 (± 0.7)	3.6 (**± **0.3)	3.3 (**± **0.2)
Sense of common humanity	3.0 (**± **0.3)	3.3 (± 0.4)	3.2 (**± **0.3)	3.3 (**± **0.3)
Isolation	3.3 (**± **0.3)	2.6 (± 0.4)	2.3 (**± **0.4)	2.8 (**± **0.2)
Mindfulness	3.0 (**± **0.2)	3.1 (± 0.3)	3.5 (**± **0.4)	3.9 (**± **0.2)
Over-identified	3.7 (**± **0.2)	3.3 (± 0.5)	2.8 (**± **0.2)	2.8 (**± **0.3)
Mindfulness/FFMQ-BR	117.7 (**± **2.9)	115.2 (± 5.4)	119.7 (**± **3.9)	125.6 (**± **4.2)
Nonjudging of inner experience	25.1 (**± **2.3)	21.2 (± 4.2)	20.6 (**± **1.8)	20.2 (**± **0.8)
Acting with awareness -autopilot	15.4 (**± **1.5)	12.5 (± 2.0)	14.9 (**± **2.2)	12.5 (**± **1.8)
Observing	24.7 (**± **2.1)	28.5 (± 3.0)	26.5 (**± **2.1)	29.9 (**± **1.9)
Describing – positive	17.4 (**± **1.5)	18.0 (± 2.3)	18.8 (**± **1.7)	19.6 (**± **0.9)
Describing—negative	7.0 (**± **1.0)	6.5 (± 1.1)	8.9 (**± **1.7)	6.3 (**± **1.1)
Nonreactivity to inner experience	17.5 (**± **1.6)	19.5 (± 1.6)	20.6 (**± **2.1)	22.2 (**± **2.2)
Acting with awareness—distraction	10.6 (**± **0.9)	9.0 (± 1.7)	7.3 (**± **0.6)	9.1 (**± **1.3)

T1: baseline, T2: post-intervention, T3: 3-month follow-up. BES: Binge Eating Scale; SCS: Self-compassion scale; FFMQ-BR: Five Facets Mindfulness Questionnaire-BRAZIL; BMI: Body Mass Index

## Discussion

Our study demonstrated that the MB-EAT intervention, when combined with elements of the MSC program, in its current format, did not meet the minimum feasibility criteria, particularly regarding adherence, session attendance, and data completeness. However, despite the low values of the feasibility indicators, preliminary suggestive improvements in self-compassion, mindfulness, and binge eating were observed among completers. In addition, the women maintained their weight during the intervention.

The dropout rate in the present study was 60% and overall attendance was 41.7%. These rates are comparable to those reported in mindfulness-based interventions conducted with post-bariatric patients, such as Wnuk et al. [26], who reported a dropout rate of approximately 42%, and Chacko et al. [25], with a dropout rate of 52.2%. High attrition rates have also been reported in psychosocial interventions targeting individuals with binge eating disorder, including behavioral and mindfulness-based programs, where dropout rates frequently range from approximately 40% to 60% [45, 46]. This pattern suggests that retention challenges are common among individuals with a history of MBS and dysregulated eating behaviors. Individuals with patterns such as binge eating or emotional eating often face greater engagement difficulties, particularly in interventions involving self-reflection and emotional exposure [45], which may lead to avoidance of internally discomforting experiences and contribute to early withdrawal. Importantly, avoidance of internally discomforting experiences should not be interpreted as a general limitation of mindfulness-based interventions, which are typically well tolerated across clinical populations, but rather as a population-specific challenge in the context of post-bariatric patients with eating-related dysregulation [47]. Taken together, these findings suggest that high dropout rates are a common challenge across delivered psychosocial interventions for binge eating and related eating dysregulation.

The hypothesis that the online format and the nature of the approach impacted feasibility is also supported by evidence from remote mindfulness-based interventions. For instance, Pérez et al. [46] evaluated an online intervention combining MB-EAT and behavioral counseling and reported a 58% dropout rate. The remote format may attenuate the early development of therapeutic rapport and group cohesion, and reduce perceived accountability, while domestic and occupational demands compete for participants’ attention, factors that may facilitate attrition particularly during the initial weeks, before group norms, trust, and practice routines are established.

These data provide important insights into which aspects of the protocol need to be improved to enhance the effectiveness and applicability of the intervention. Early dropout (in the initial weeks) may suggest weaknesses in developing therapeutic rapport and group engagement during the early stages of the intervention [48]. It is worth noting that an introductory session was held before the start of the intervention, aiming to align expectations about the protocol, present the program content and benefits, and emphasize the importance of group commitment. However, this strategy was not effective in preventing early dropout.

These findings are consistent with the informal facilitator feedback indicating a mismatch between participants' expectations (e.g., traditional treatment methods such as diets, medication) and the content offered in a mindfulness-based intervention, reinforcing the possibility that the introductory session may not have been sufficient to realign these prior expectations. Future studies would benefit from incorporating a qualitative component to better understand participants’ expectations, lived experiences, and engagement challenges, thereby informing strategies to improve adherence. This mismatch should be understood in light of the biopsychosocial context of women with weight regain after MBS. Previous studies have shown that this group often experiences intense feelings of shame, failure, and self-depreciation when weight regain occurs, which can negatively affect motivation and engagement in psychosocial approaches [14, 15]. Moreover, these women face aesthetic and emotional pressures and frequently attribute a transformative, definitive role to the surgery, both physically and socially [14]. When expected outcomes are not achieved, individuals may experience emotional withdrawal and increased avoidance behaviors, which can hinder engagement with supportive therapeutic approaches such as mindfulness and self-compassion. It is also essential to consider that women with a history of eating disorders or emotional dysregulation, especially after surgery, struggle more to engage in programs requiring self-reflection or emotional exposure [26, 27]. Additionally, the remote format may have weakened interpersonal connections and made dropout easier [49].

While the recruitment rate indicates moderate success in attracting eligible participants, it also highlights potential barriers to recruitment in this population. This is a hard-to-reach sample [50], due to the specific profile (women experiencing weight regain after MBS) and to the demands of the intervention protocol itself, which may have limited alignment with the proposed approach or created logistical challenges to participation.

In this sense, the feasibility findings point to areas in the intervention protocol that can be adjusted to increase participant retention, including the recruitment method, expectation alignment before the intervention begins, measures such as reducing the number and length of sessions, offering greater flexibility in session scheduling (e.g., spacing sessions rather than holding them weekly), and referring participants through multidisciplinary clinical teams may support adherence.

Despite the challenges described above, it is essential to highlight that the participants who completed the intervention showed a high average session attendance rate (87.5%), indicating good engagement and reporting positive experiences with the content, particularly concerning improvements in eating behavior and self-compassion development. This suggests that the intervention holds promise, especially among women who are already more ready for this approach.

It is important to highlight that binge eating is the most prevalent component of eating disorders among bariatric patients [51, 52]. Research conducted with people after MBS has shown that increased awareness and attention during eating reduced binge eating episodes [53, 54]. Our study observed suggestive results for reducing binge eating scores from pre- to post-intervention MB-EAT/MSC. These outcomes may be related to participants' opportunity to observe their experiences without judgment or criticism, thereby preventing dysfunctional eating behaviors and promoting alternative strategies for coping with negative emotions [55]. However, these findings were not maintained at follow-up; three months later, BES scores had increased. In post-bariatric populations, binge eating is often better conceptualized in terms of loss-of-control eating and associated distress, as physiological restriction may limit the consumption of objectively large amounts of food. In this context, the Binge Eating Scale should be interpreted in this context as a measure of symptom severity rather than as a diagnostic tool, and its scores must be considered in light of the anatomical and physiological changes imposed by surgery. These findings are consistent with Wnuk et al. [26], who observed improvements in binge eating after the MB-EAT intervention, but with benefits that were not sustained over time. Although the reduction in binge eating improvements at the 3-month follow-up is disappointing, similar attenuation of treatment effects over time has been reported in interventions targeting binge eating in post-bariatric populations and related clinical contexts [25, 26, 45, 46], as well as in non-surgical populations receiving psychological treatments for binge eating [56]. Nevertheless, it is important to emphasize that, despite the increase in BES scores between T2 and T3, there was still a suggestive improvement when comparing baseline values to those at 3-month follow-up. Taken together, these findings suggest that patients with binge eating may require long-term or ongoing support to sustain potential changes in dysfunctional eating behavior, and that improving the durability of treatment gains remains a recognized and ongoing goal within the broader eating disorder treatment field.

Many individuals go through daily life on “autopilot,” relying on habitual patterns that reduce awareness of the present moment and limit flexibility in responding to everyday situations [57, 58]. Mindfulness-based interventions (MBIs) promote changes in individuals’ perspectives on their internal experiences — Cognitive Flexibility [57, 58]. Such changes were observed in the present study, with preliminary evidence of improvements in mindfulness-related outcomes. Notably, there was a decrease in the FFMQ-BR subscale “acting with awareness – distraction” following the intervention and an increase in the “Mindfulness” and the “Nonreactivity to Inner Experience” subscales over the course of the study. These patterns may reflect greater familiarity with and integration of mindfulness practices into daily life. A possible explanation, as described in the literature, is that mindfulness practice is associated with neurocognitive and behavioral changes involving brain regions related to self-awareness and self-regulation, with effects that may persist beyond the formal practice period [59]. The internalization of these practices may contribute to the maintenance of therapeutic changes over time, even gradually or later [60]. In this context, practitioners may learn to accept thoughts, feelings, and bodily sensations, recognizing thoughts as mental events rather than absolute truths, a process known as cognitive defusion and increased psychological flexibility. This reduction in automatic reactions (distraction) is associated with decreased repetitive negative thinking [61, 62].

Mindfulness practice promotes more self-compassionate responses. Individuals with higher levels of self-compassion tend to show lower self-criticism, less body dissatisfaction, greater motivation to adopt healthy behaviors, and better emotional regulation than those with lower self-compassion levels [63, 64]. In the present study, we observed a trend toward a decrease in the “over-identification” and “isolation” subscales after the intervention and an increase in mindfulness levels. A plausible explanation is that individuals with higher self-compassion tend to ruminate less on their difficulties and are more capable of embracing their imperfections with kindness and understanding, which may help break cycles of self-criticism and negativity [63]. Beyond its general psychological benefits, self-compassion may play an important role among individuals who have undergone MBS, who often face challenges related to weight regulation, body image, and emotional adjustment during the postoperative period [65, 66]. Cultivating self-compassion may help them respond to setbacks with greater understanding and less self-criticism, reducing avoidance and promoting re-engagement in health-promoting behaviors. Higher self-compassion has been associated with lower disordered eating, greater body image satisfaction, and better emotional regulation [19, 67]. Moreover, self-compassion has been linked to improved psychological functioning and well-being in this population, suggesting that it may buffer the effects of internalized stigma and support sustainable behavioral change [27]. Although emerging evidence indicates potential benefits of self-compassion for weight management, available findings remain limited and heterogeneous, underscoring the need for more rigorous investigation [65].

Another preliminary finding was a trend toward reductions in both weight and BMI at the 3-month follow-up compared with baseline. These patterns contrast with the findings of Wnuk et al. [26] and Chacko et al. [25], who did not observe weight or BMI changes among their participants immediately after the intervention or at follow-up. However, it is important to emphasize that weight loss is not the primary goal of mindfulness-based interventions. Nonetheless, weight-related changes may be indirect effects of improved relationships with food and increased awareness of internal and external cues related to hunger, satiety, and emotional eating [60]. A recent systematic review highlighted the variability in weight-related outcomes across studies involving individuals who underwent MBS and participated in mindfulness-based interventions. While some studies reported modest or significant weight loss, others found stability over time. Differences in assessment tools, sample characteristics, and the lack of control groups in many of these studies may contribute to this variability and limit more conclusive interpretations regarding the potential effects of these interventions [68].

This study has some limitations that should be acknowledged. The small sample size allows for a descriptive analysis of the results, preventing statistical inferences. The specific characteristics of the sample, composed exclusively of women in the post-MBS period who experienced weight regain and reported binge eating episodes, may limit the generalizability of the findings. Furthermore, we did not collect data on participants’ adherence to nutritional follow-up or multidisciplinary care, factors that may influence postoperative outcomes. Nor did we include psychiatric and psychological assessment during follow-up, which could have provided valuable information on the emergence of new psychopathology, or on psychological constructs, such as alexithymia and emotional regulation difficulties, that may influence engagement, adherence, and potentially be related to weight regain. Finally, we did not investigate whether preoperative binge eating symptoms evolved into other maladaptive eating behaviors, such as grazing or sweet eating, which may reflect distinct postoperative trajectories. It is important to highlight that grazing, defined as the repetitive consumption of small amounts of food over time, often outside planned meals and sometimes accompanied by a sense of loss of control [5, 69], is highly prevalent in the post-MBS context and has been associated with poorer weight outcomes [6].

However, despite the identified limitations, the data obtained in this feasibility study provide essential insights for refining the current protocol and planning future studies. The experiences reported by the participants who completed the intervention and the high attendance rate in this subgroup suggest that the program may be acceptable and relevant for women who are more ready for mindfulness- and self-compassion-based approaches. Future studies, primarily randomized clinical trials with larger samples, longer follow-up periods, and an adapted protocol that also incorporates psychiatric and psychological assessment, will be essential to evaluate the efficacy of the intervention and its applicability in diverse clinical settings. In addition, it will be important to examine other maladaptive eating behaviors beyond binge eating, such as grazing, which represents a distinct yet overlapping pattern and may influence postoperative outcomes. Taken together, these findings suggest that mindfulness-based interventions should not be dismissed in the context of post-bariatric weight regain, but rather require careful adaptation and feasibility-informed design to be clinically sustainable and meaningful for this population.

In conclusion, the current form of the MB-EAT/MSC intervention appears unfeasible, particularly regarding adherence, session attendance, and protocol sustainability, emphasizing the need for structural and strategic adjustments to enhance feasibility in this population. Specifically, future studies should consider strengthening early therapeutic rapport and group engagement, providing clearer expectation alignment prior to enrollment, reducing the number or duration of sessions, increasing scheduling flexibility, involving multidisciplinary referral pathways to support adherence, and reassessing the online delivery format to foster interpersonal connection. Moreover, despite these feasibility challenges, the intervention showed preliminary benefits in self-compassion, mindfulness, and binge eating, supporting its potential relevance for further refinement and testing.

In this sense, these findings indicate that the intervention, in its current format, is not yet ready for larger-scale trials. Instead, future work should prioritize implementing the structural and strategic adjustments outlined above, followed by renewed feasibility testing to determine whether these modifications effectively improve retention, adherence, and overall viability.

## Data Availability

The data tab is found on this link: https://docs.google.com/spreadsheets/d/1gds-BltdoaklQkfSvqpZ0FwB5AwOTU87/edit?usp=sharing&ouid=103601650807872773658&rtpof=true&sd=true.
